# A Concise Account of Dr. Gall's New Doctrine of the Brain, and the Faculties of the Mind

**Published:** 1805-10-01

**Authors:** 


### A concise Account of Dr. Gall's new Doctrine of the Brain, and the Faculties of the Mind.

( Communicated by Dr. Arneman. )

DR. GALL, of Vienna, already known to the medical world by several very valuable and ingenious medical treatises, has of late greatly increased his reputation as an ingenious and profound investigator of the operations of nature, concerning the functions of animal life, that he justly may be ranked amongst the most extraordinary men of the present age.

Dr. Gall is far from Charlatanism, neither does his theory originate from arguments formed by fancy and imaginary facts; the only rule he follows is mere and simple observation of Nature, confirmed by experience. He himself is willing to allow, that his doctrine for the present, is very far from being complete, and he is now engaged in a tour through the Continent, with the intent to display his doctrine in private lectures, to increase by these means his stock of observations, to collect the objections of the different medical men and literati, and to model more and more his assertions and the conclusions he draws from them, according to nature and general experience.

The Doctor has been for several months at Berlin, where he had the honour of delivering his doctrine before their Prussian Majesties, the Physicians of the Court, all the Medical Professors, and among them the Nestor of the present Anatomists, Dr. Walter; besides, the whole faculty, and almost every body who makes a claim to a liberal education, attended these lectures.

From thence the Doctor proceeded to Dresden and Halle, where the celebrated Anatomist Dr. Loder attended his lectures, and he intends visiting all the universities of Germany, &c. &c.

As the Doctor has not himself published an account, nor even the outlines of his doctrine, the different pamphlets published by young physicians, who attended Dr. Gall's lectures at Vienna, or saw his collection of skulls, are of very indifferent value, and for the greater part not to be depended on. We think ourselves entitled to lay a concise account of this very interesting and important doctrine before our readers, as we have several copies of the Doctor's lectures at Berlin, before us; and besides, a model of a skull formed in gypsum after the pattern the Doctor uses in his lectures for explaining his assertions;

 and and have compared this with the account of our correspondents, and several eye-witnesses.

Dr. Gall's doctrine may be divided in two parts. I. The Doctrine of the Brain. II. The Doctrine of the Skull, the Organology and Organoscopy, and Encephalognomiæ.

THE DOCTRINE OF THE BRAIN

Originated from the Doctor's numerous observations and dissection of hydrocephali interni, and from these he was led to conclude that the brain was not a pulpous substance as it is generally believed, but a mere *membrane*. In his dissections of the brain he followed not the common method, to begin the dissection from the cortical substance, downwards to the basis encephali. On the contrary, he always made a beginning with the anatomy of the spinal marrow, and so upwards. In this way he found that the structure of the brain is very different from what is the general opinion of anatomists. By many years indefatigable labour and observation, the Doctor found, that animals of the most simple organisation have only filaments of nerves dispersed through the body. In the next class of animals he found the trunk of the spinal marrow formed. The more compleat classes of animals are endowed with nerves rising from the spinal marrow. In the highest classes the spinal marrow is double, like all other organs on which animal life depends; and a double series of nerves rises from the spinal marrow. *The spinal marrow forms the brain and all other nerves without exception*, although it may appear that they take their origin from the brain. There is no nervous substance or medulla nervea, but the nerves consist only of filaments, &c.

All nerves rising from the spinal marrow, increase in size in proportion as they enter the cranium and take their course to the surface of the brain; *they all pass on their way through nervous knots, ganglia.* These ganglia of the brain, contain a texture or expansion of nerves, mixed with a substance like the cortical substance of the brain, and may be considered as the matrix, or serving for their nourishment. Of the nerves rising from the spinal marrow, *eight pair hitherto are known.* The Doctor demonstrates them as follows: From the upper part of the spinal marrow, and the medulla oblongata, are formed on each side the *nervus accessorius*, and *nervus oculomotorius*, and as their common *ganglion* may be considered the *corpus olivare*. More towards the centre of the medulla oblongata we find *the nerves forming the cerebellum*, or, as the anatomists mists call them, *processus cerebelli ad medullam oblongatam,* the ganglion of these fascicles of nerves is the *corpus ciliare,* situated in the *arbor vitæ* of the *cerebellum.* Next to these we find the *acoustic nerves,* the *optic nerves,* and the *nervi olfactorii;* all these pass through a ganglion, as by a minute dissection easily appears.

The most important of all the eight pair of nerves is what was hitherto called the *pyramids,* this fascicle of nerves is *the origin of the cerebrum,* or the *hemispherii cerebri.* The Doctor proves this by observing, *that the size of the hemispheres of the cerebrum always is proportionate to the size of the pyramids;* besides the pyramids take their course uninterruptedly to the surface of the hemispheres. The pyramids have allowed to them *two ganglia,* the first is the *pons Varolii,* or *protuberantia annularis Willisii,* and the second the *ganglion cerebri,* which appears in dissecting the *lobum cerebri* next the *fossa sylvii.* These nervous strings form the *gyri cerebri.*

It would lead too far to follow the Doctor through his very minute dissections and enumerations of all the filaments of nerves, by which he traces his assertions, we shall leave this to the attention of anatomists.

It has been said above, that, according to Dr. Gall, all nerves take their origin from the spinal marrow, eight pair of which enter the cranium; these he calls *entering nerves.* But on a minute examination, we find some nervous filaments returning from the brain. The Doctor considers these as a different species of nerves, and calls them *returning,* or *retrograde* nerves. *They take their rise from the cortical substance* that constitutes and surrounds the ends of the first species of entering nerves. They increase by joining towards the spinal marrow, and they do not pass through ganglia; but they form, as the Doctor observed, *commissuræ;* for instance, the commissura of the *retrograde acoustic nerve* is situated behind and under the pons Varolii; the commissura of the *nervi olfactorii,* between the ganglion belonging to these nerves; the commissura of the *retrograde nerves of the cerebellum* is in the pons Varolii itself. The *retrograding nerves of the cerebrum* have more commissura belonging to them; the first is the corpus callosum; the second the commissura anterior of the lobus cerebri bordering on the optic nerves, the *septum pelucidum* is a continuation of this, &c.

Besides these two species of nerves, we observe a very delicate and subtle substance of nerves, passing from the spinal marrow, through the very middle, upwards, and

2 ending ending in the raphe Lavoisii on the corpus callosum. This substance, Dr. Gall considers as the *uniting medium between the double organs* through the nervous system.

### The Organology.

Laying down as a basis, that every string of nerves the ganglion of the cerebrum consists of, forms the different gyri cerebri; and being proved by experience, *that the state and growth of the brain forms and shapes the bony part, or the skull,* the Doctor was led to conclude, that the different elevations and depressions we observe in a manifold manner by comparing a number of heads in living persons, may be considered like the seat of so many different organs, proving and expressing the faculties of the mind, any animal or human being is endowed with by Nature.

As it cannot be denied that every eminent talent is a gift of nature, cultivated by education and art; and the faculties and powers of the mind, being so very differently distributed; the Doctor obviates every objection that his doctrine may lead to promote materialism. The anatomists and physiologists almost of every age, endeavoured to investigate the seat of the soul, and to find out the very spot the different faculties of the mind are resident. Dr. Gall, on the contrary, considers the brain, not as the common organ of the soul, but as *an assembly* or an *aggregation of the different organs.*

These *organs,* according to the Doctor, *manifest themselves* to the touch, or the eye-sight, *by elevations and prominences of the outer part of the skull,* and they are uniformly to be found in animals as well as in the human race. So, for instance, we find a striking likeness in the formation of the skull of rapacious quadrupeds and birds; the skull of domestic animals, or of such that are noted for mildness of temper, is totally different. This likeness the Doctor traces through all classes of animals, and as far as his vast and deep experience confirms it, draws a comparison with mankind.

This doctrine may be considered therefore, for the future, of the most important use in education. The different organs undergoing an evolution, depending partly on the general growth of the brain, partly on the different periods of age; in old age, when the faculties of the mind decrease, the condition of the brain and the shape of the skull becoming very different, and undergoing visible changes. It may be of the greatest importance to promote mote and regulate the growth and forwardness of different organs by education and moral impressions, mean while the other pernicious organs must be forcibly depressed and retarded. The organs being independent of each other, yet among the neighbouring organs there exists a consentient association; and by rousing the energy of one organ, all the neighbouring parts may be affected and drawn into action. These organs, of course, in people of common faculties, are not very prominent; most conspicuously we find them in persons of brilliant talents, great geniuses, and in those whose minds are deranged. Dr. Gall points out *thirty-two* organs in the following order.

1. *The organ of life*, vis vitalis. Its seat is close to the *foramen magnum ossis occipitis*. All persons who attained a great age, as the Doctor observed, have a large foramen occipitis. This we find wonderfully confirmed in all animals that are noted for their longevity; the elephant, the eagle, the parrot, the swan, the raven, the carp, &c.— Wounding this part, instantaneous and absolute death is the consequence.

2. *The organ for preserving life*, wish for life. There are persons to whom life is very indifferent, though they live in the greatest affluence. On the contrary, we find wretches in dungeons, loaden with chains, who are most wonderfully attached to life. This organ is to be found near the processus cuneiformis ossis occipitis. It would be worth while to examine the heads of suicides.

3. *The organ of sexual love*. Near the foramen ossis occipitis, underneath the linea semicircularis inferior. It is double, and in nerves of the ars amandi and in the nymphomania, like a protuberance on each side of the head. Dr. Gall places the organ of sexual love in the cerebellum. In all animals that excel in this quality, viz. the stallion, the bull, these protuberances likewise are in very great perfection, therefore the neck is much broader, and the occiput appears larger. This is the same case with the monkey, the cock, the sparrow. On the contrary, in all castrated animals, the gelding, the ox, the human castrati, these protuberances are wanting or much less, and the neck is much thinner. It is known to the faculty, that constant painful sensation in this part of the brain is complained of by all persons suffering by the consequences of onany and debauchery. Mules have a very small cerebellum, &c.

4. *The organ for love of children*, and in animals of *their young* ones. This organ in general is in greater perfection in in women, and particularly in those who are noted for their maternal attachment to children. In monkeys it is likewise very strongly marked. Also in people who put their greatest happiness in domestic life. It is very interesting to compare the heads of animals where the male leaves the young ones soon after birth, with those that shew the most tender attachment for their offspring. Likewise the head of those birds who leave their eggs to others to hatch, viz. the cuckoo. This organ is situated on that part of the occiput, inclosed by the margines lambdoidalis and the protuberantia occipitalis externa.

5. *The organ for friendly attachment, love, sociality, faithfulness,* is situated a little above the last mentioned organ, on both sides; or just above the sutura lambdoidea. It is in the highest perfection in people noted for their friendship to each other. The Doctor observed it likewise in malefactors, who sustained the greatest tortures merely to save their companions. Among animals it is very conspicuous, and particularly in some species of the dog kind, viz. the barbet.

6. *The organ for delight in fighting, rioting, quarelling.* Formerly the Doctor called this the *organ for courage,* but this expression is not wide enough. On the head of the Austrian General Wurmbser, the Doctor found it excessively large, and likewise among the mob, fishwomen, &c. It would be worth while to examine the heads of great boxers. Among the animal tribe we observe it likewise, particularly in some species of dogs, the lion, &c. Great cowards and timid animals, viz. the hare, have a depression at this spot of the head, or the protuberance is scarcely perceptible.

7. *The organ for the propensity of murdering.* It is situated on each side of the head, before the last mentioned organ, but more towards the meatus auditorius, just above the margo temporalis. In granivorous animals it is wanting. On the contrary, all carnivorous animals, and of course mankind, possess this organ in greater or less proportion. In the tyger we find it in the greatest perfection. The Doctor observed it likewise very eminent in some great warriors, in people that have an invincible propensity of becoming butchers, hangmen, &c. Also in those unfortunate beings, we find instances of in criminal trials, who murder their fellow creatures without any particular motive, but merely from an internal instinct.

8. *The organ for cunning.* In general, the forehead of persons noted for this organ is uncommonly broad. It is situated situated about two inches above the meatus auditorius, on the angulus sphænoidalis on each side of the head; and in the greatest perfection the Doctor saw it on the head of the great Frederic of Prussia, Prince Kaunitz, several great statesmen, eminent theatrical performers, poets, &c. Among animals, the head of the panther, the fox, the cat, the greyhound, and several birds, &c. prove this assertion.

9. *The organ for stealing*, is situated close before the last organ, more towards the sutura coronalis, on both sides of the head. This disgracing organ is undoubtedly proved by experience and observation. Among animals, the magpye, the crow, the cat, the monkey, &c. are instances. And likewise we find among the human species, individuals, who very far from want or necessity, feel an irresistible propensity to steal. The first King of Sardinia, Victor, was noted for stealing. Among felons and robbers, the Doctor found this organ in the greatest perfection.

10. *The organ for artificial objects, fine arts*, situated on the os frontis, just behind the apophysis jugalis, is likewise double. The Doctor found it most extraordinary on the head of Raphael; besid s, in great mechanics; women that are noted for fine work, millinery, &c. Among animals, in the beaver.

11. *The organ for music.* Above the angulus externus of the orbita, on the anterior part of the linea semicircu-laris ossis frontis, on each side of the head. The front becomes broader or longer when this organ is in the highest perfection. This we find confirmed in all great musical performers and connoiseurs; viz. the head of Viotti, Mozart, Gluck, the Emperor Joseph, Haydn, Cherubini, &c. Among birds, the whole tribe of singing birds are in possession of this organ, principally the nightingale, and some species of water birds noted for imitating the song of almost all other birds, (mock birds). In other animals this organ is totally wanting, viz. the monkey. Also in people who are averse to music it is scarcely perceptible.

12. *The organ for calculating, arithmetic*, has its seat on the upper corner of the orbita, in the fossa glandulae lacrymalis ossis frontis, on both sides. Animals do not possess this organ. The Doctor very seldom observed it in negroes, but in the greatest eminence in calculators and mathematicians.

13. *The organ for the remembrance of names, words.* It is situated deeply in the orbita; people, therefore, endowed with this faculty, always have prominent eyes. Some literati, literati, compilers, possessors of large collections, &c. not being able to make a proper use of their great stock of knowledge, belong to this class.

14. *The organ for learning languages, the phylological talent,* is quite different from the last mentioned organ; it is situated on the anterior corner of the orbita. When in the highest perfection the eye becomes more depressed; this, for instance, is the case with the great scholar Wolf, editor of Homer, with Montaigne, and others. No animal possesses this organ.

15. *The organ for local remembrance,* on both sides of the root of the nose, towards the middle of the arcus superciliaris. When in the highest perfection, visible protuberances are formed. This we observe in eminent travellers, particularly those who are very accurate in local descriptions, viz. Captain Cooke; in great engineers; and the Doctor observed it eminently on the head of the famous Austrian General Mack; also in great astronomers, painters of landscapes, &c.

16. *The organ for colouring,* begins from the middle of the arcus superciliaris, and ends outwardly towards the upper corner of the orbita. Some great painters, admired for their talent of colouring, and the brilliancy of their colours, belong to this class, viz. Titian, Corregio, Van Dyck, &c.

17. *The organ for remembering persons.* There are people endowed with such a wonderful memory, that, after years have elapsed, instantly remember persons they have seen but once in their life, or met with only by accident. This organ is situated below the foramen supraorbitale, towards the nose, and appears double. When in perfection, the eye is a little depressed towards the nose, and has a squinting appearance.

18. *The organ for circumspection,* manifests itself by a prominence more towards the temples, about the linea semicircularis ossis bregmatis, on both sides. In all animals prosecuting their prey during night, it is to be met with; particularly the owl, the marder; also the fallow deer, the goat of the Alps, &c. who are known for being very circumspect. In children in general it is strongly marked, also in very considerate, cautious, and circumspect people. The head of Frenchmen, as the Doctor observes, is at this spot more pointed. In all inconsiderate and flighty people; for instance, beggars by profession, it is very imperfect, or scarcely to be distinguished.

19. *The organ for liberality.* In all people of liberal sentisentiments, the front is softly and pleasingly rounded. Misers have always a visible depression across the front.

20. *The organ for comparative ingenuity, sagacity,* appears single, like a conical protuberance at the middle of the os frontis. In clergymen known for talents of eloquence, and great orators, it is in greatest perfection.

21. *The organ for metaphysical ingenuity,* is situated on each side of the last mentioned organ. When in pre-eminence, it almost forms a triangular protuberance, viz. at the head of the great Philosopher Kant.

22. *The organ for wit.* At the very spot where the tubera frontalia are situated, on both sides. In great wits the tubera become quite prominent, viz. Voltaire.

23. *The organ for observing genius,* more towards the temples, and above the tubera frontis. Almost all children are instances of it.

24. *The organ for representing genius.* A protuberance at the upper part of the front. All great theatrical performers, viz. Garrick, possess this organ in greatest perfection; likewise dramatical authors and eminent poets.

25. *The organ for goodness,* about the middle of the front, between the tubera frontalia. It appears single, both organs being closely connected. People who distinguish themselves by a barbarous and cruel behaviour, have a remarkable depression instead; for instance, Robespierre, Nero. Also all rapacious animals, the tyger, the hyena, the eagle, some species of dogs, the crocodile. In horses and cows, this depression is a certain proof of their ill temper, well known to dealers in these animals.

26. *The organ for religious propensity, theosophy.* This is the highest of all organs, at the very summit of the front, above the last mentioned organ. All devout, religious, and fanatical people possess this organ in pre-eminence. The paintings of our Saviour and the Madona, may be considered as instances.

27. *The organ for firmness of mind, constancy.* At the spot where the anguli frontales ossium bregmatis meet together. In inconstant and whimsical people, we observe a depression instead. It is strongly expressed in great mechanics, who with the utmost perseverance engage themselves for a number of years in constructing the most complicated machines; likewise those who prosecute law-suits for twenty or thirty years.

28. *The organ for haughtiness,* appears at the middle of the sutura sagittalis, singly; both organs neighbouring each other. All proud and haughty people, madmen, &c.

of of this class, shew a very remarkable protuberance at their head. Animals that climb the highest mountains, all birds that are fond of high places, viz. the eagle, have this organ more elaborate than others dwelling in lower regions.

29. *The organ for celebrity, fame, vanity*, situated on each side of the organ for haughtiness, and is doubly marked. In female heads the Doctor observed it more frequent than in men.

30. *The organ for veracity, truth.* Above the point of the sutura lambdoidalis. If a depression is observed instead, we may draw the conclusion for a story-telling temper. Daily experience, and knowledge of mankind, prove clearly, that want of veracity is, sometimes, not owing to education or habit, but founded in the organology of the brain.

31, 32. The functions of these two organs are not yet ascertained by the Doctor. He sometimes observed in examining skulls, above the meatus auditorius, a very remarkable protuberance; and in others, this part more flat and depressed. He is inclined to think, that they may belong to the organ of hearing, and consequently constitute *the organ for accurate and just hearing*, of which, great musical performers and virtuosos are possessed.

Comparing and surveying this whole series of different organs, distributed on the head of mankind, we may rank them in three classes. The first comprehends the Organs for preserving and maintaining Life; the second, the Organs for the different Propensities, Affections, and Passions; the third, the Organs for the intellectual and moral Faculties.

The human species unites all the organs, without exception, that are met with in animals; and it would be possible, by properly placing the different human organs, to form and represent all the single organs that animals possess. On the contrary, all the organs which animals are deprived of, are situated in the front of men; and the formation and evolution of this part of the brain, totally different, and for the greater part wanting in animals, constitutes the great line that intervenes between the animal tribe in general and the human species.

